# Uncharted territory: assessing antibiotic adverse drug events from walk-in clinics at an academic healthcare system

**DOI:** 10.1017/ash.2026.10356

**Published:** 2026-04-17

**Authors:** Nishant Patel, Michael Zou, Emily Nardone, Retha Thomas, Angela Zuill, Claude Shackelford, Minhau Zhang, Sharon Onguti, Milner Staub

**Affiliations:** Division of Infectious Diseases, Department of Medicine, Vanderbilt University Medical Center, USA

## Abstract

Outpatient antibiotic ADE rates remain poorly defined due to limited tracking methods. We evaluated the use of ICD-10 billing codes, voluntary reporting, and manual review approaches to identify outpatient antibiotic ADEs with variable results. Improved, systematic methods are needed to accurately track antibiotic ADEs and support Antimicrobial Stewardship efforts.

## Introduction

An estimated 30%–50% of prescribed outpatient antibiotics are unnecessary or inappropriate.^1^ Antibiotic overuse is associated with increased patient harm.^2^ Prospective audit and feedback (PAF) effectively reduces antibiotic prescribing.^3,4^ Patient outcomes-centered provider feedback, like adverse drug effects (ADEs) feedback, effectively changes behavior.^5,6^ However, few PAF reports provide antibiotic ADE feedback.

At Vanderbilt University Medical Center (VUMC), outpatient adult antibiotic-associated ADE rates are unknown. We aimed to: (1) assess antibiotic ADEs via retrospective electronic data extraction using billing codes and evaluate process challenges; (2) determine ADE rates, antibiotic appropriateness, and associated billing codes via manual chart review at eight VUMC walk-in clinics (WICs); and (3) pilot a system to log patient calls and return visits for ADEs to enhance data capture.

## Methods

### Defining and capturing antibiotic ADEs via international classification of diseases (ICD)-10 codes

We attempted to automate the identification of antibiotic-associated ADEs via electronic medical record (EMR) billing data. Based on experience, three authors (NP, MS, SO) compiled 134 ICD-10 codes representing potential common side effects from antibiotics experienced by adults (Table 1). Adult outpatient and emergency department encounters were included, excluding those that led to admission. Criteria evolved across five iterative exploratory attempts resulting in manipulation of time frame, clinics included, and ICD-10 codes to attempt to optimize yield. Manual chart review was performed on >10% of encounters (selected via a random number generator) for attempts 1, 2, and 4, and on all encounters for attempts 3 and 5 (Table 2).

**Table 1.** ICD-10 codes classified by organ system

Organ system/category	ICD-10 code and description
Gastrointestinal	**A04.7 ENTEROCOLITIS DUE TO *CLOSTRIDIUM DIFFICILE*** **A04.71 ENTEROCOLITIS DUE TO *CLOSTRIDIUM DIFFICILE, *RECURRENT** **A04.72 ENTEROCOLITIS DUE TO *CLOSTRIDIUM DIFFICILE*, NOT SPECIFIED AS RECURRENT** ***B37.81 Candidal esophagitis*** R11.0 Nausea R11.10 Vomiting, unspecified R11.11 Vomiting without nausea R11.2 Nausea with vomiting, unspecified R19.4 Change in bowel habit R19.7 Diarrhea, unspecified R10.9 Unspecified abdominal pain R10.10 Upper abdominal pain, unspecified R10.30 Lower abdominal pain, unspecified R10.84 Generalized abdominal pain R14.1 Gas pain R10.33 Periumbilical abdominal pain R10.11 Right upper quadrant pain R10.12 Left upper quadrant pain R10.31 Right lower quadrant pain R10.32 Left lower quadrant pain R10.13 Epigastric pain R14.0 Abdominal distension (gaseous) K30 Functional dyspepsia K71 Toxic liver disease K71.0 Toxic liver disease with cholestasis K71.1 Toxic liver disease with hepatic necrosis K71.10 Toxic liver disease with hepatic necrosis w/o coma K71.11 Toxic liver disease with hepatic necrosis w/o coma K71.2 Toxic liver disease with acute hepatitis K71.6 Toxic liver disease with hepatitis, not elsewhere classified K71.8 Toxic liver disease with other disorders of liver K71.9 Toxic liver disease, unspecified R94.5 Abnormal results of liver function studies
Hematologic	D61.1 Drug-induced aplastic anemia D61.811 Other drug-induced pancytopenia D61.2 Aplastic anemia due to other external agents D61.818 Other pancytopenia D61.9 Aplastic anemia, unspecified D64.9 Anemia, unspecified D69.59 Other secondary thrombocytopenia D69.6 Thrombocytopenia, unspecified D72.810 Lymphocytopenia D72.818 Other decreased white blood cell count D72.819 Decreased white blood cell count, unspecified D72.89 Other specified disorders of white blood cells D61.811 Other drug-induced pancytopenia D61.818 Other pancytopenia
Renal	N14 Drug- and heavy-metal-induced tubulo-interstitial and tubular conditions N14.1 Nephropathy induced by other drugs, medicaments and biological substances N14.2 Nephropathy induced by unspecified drug, medicament or biological substance N17 Acute renal failure N17.0 Acute renal failure with tubular necrosis N17.1 Acute renal failure with acute cortical necrosis N17.2 Acute renal failure with medullary necrosis N17.8 Other acute renal failure N17.9 Acute renal failure, unspecified N19 Unspecified kidney failure R94.4 Abnormal results of kidney function studies
Neurologic	R42 Dizziness and giddiness R51.0 Headache with orthostatic component, not elsewhere classified R51.9 Headache, unspecified G44.40 Drug-induced headache, not elsewhere classified, not intractable G44.41 Drug-induced headache, not elsewhere classified, intractable G44.89 Other headache syndrome
Dermatologic	***B37.2 Candidiasis of skin and nail*** L50 Urticaria L50.0 Allergic urticaria L50.8 Other urticaria L50.9 Urticaria, unspecified L51 Erythema multiforme L51.0 Nonbullous erythema multiforme L51.1 Stevens-Johnson syndrome L51.2 Toxic epidermal necrolysis L51.3 Stevens-Johnson syndrome toxic epidermal necrolysis overlap syndrome L51.8 Other erythema multiforme L51.9 Erythema multiforme, unspecified R21 Rash and other nonspecific skin eruption D72.12 Drug rash with eosinophilia and systemic symptoms syndrome L27.0 Generalized skin eruption due to drugs and medicaments taken internally
Cardiac	R07.1 Chest pain on breathing R07.2 Precordial pain R07.81 Pleurodynia R07.89 Other chest pain R07.9 Chest pain, unspecified
Pulmonary	R06.00 Dyspnea, unspecified R06.02 Shortness of breath R06.09 Other forms of dyspnea R06.2 Wheezing R06.89 Other abnormalities of breathing R06.9 Unspecified abnormalities of breathing
Genitourinary	***B37.3 Candidiasis of vulva and vagina*** ***B37.31 Acute candidiasis of vulva and vagina*** ***B37.4 Candidiasis of other urogenital sites*** ***B37.41 Candidal cystitis and urethritis*** ***B37.42 Candidal balanitis*** ***B37.49 Other urogenital Candidiasis***
General/Other	***B37 Candidiasis*** ***B37.0 Candidal stomatitis*** ***B37.89 Other sites of candidiasis*** ***B37.9 Candidiasis, unspecified*** R94.8 Abnormal results of function studies of other organs and systems R53.1 Weakness R53.81 Other malaise R53.83 Other fatigue M62.81 Muscle weakness (generalized) R68.83 Chills (without fever) R50.9 Fever, unspecified R50.2 Drug-induced fever
Medication-related	T36.95XA Adverse effect of unspecified systemic antibiotic, initial encounter T36.95XS Adverse effect of unspecified systemic antibiotic, sequela T36.95XD Adverse effect of unspecified systemic antibiotic, subsequent encounter T36.8X5A Adverse effect of other systemic antibiotics, initial encounter T36.8X5S Adverse effect of other systemic antibiotics, sequela T36.8X5D Adverse effect of other systemic antibiotics, subsequent encounter T36.0X5A Adverse effect of penicillins, initial encounter T36.0X5S Adverse effect of penicillins, sequela T36.0X5D Adverse effect of penicillins, subsequent encounter T36.1X5A Adverse effect of cephalosporins and other beta-lactam antibiotics, initial encounter T36.1X5S Adverse effect of cephalosporins and other beta-lactam antibiotics, sequela T36.1X5D Adverse effect of cephalosporins and other beta-lactam antibiotics, subsequent encounter T36.4X5A Adverse effect of tetracyclines, initial encounter T36.4X5S Adverse effect of tetracyclines, sequela T36.4X5D Adverse effect of tetracyclines, subsequent encounter T37.0X5A Adverse effect of sulfonamides, initial encounter T37.0X5S Adverse effect of sulfonamides, sequela T37.0X5D Adverse effect of sulfonamides, subsequent encounter T36.3X5A Adverse effect of macrolides, initial encounter T36.3X5S Adverse effect of macrolides, sequela T36.3X5D Adverse effect of macrolides, subsequent encounter T88.6XXA Anaphylactic reaction due to adverse effect of correct drug or medicament properly administered, initial encounter T88.7XXA Unspecified adverse effect of drug or medicament, initial encounter T88.7XXS Unspecified adverse effect of drug or medicament, sequela T88.7XXD Unspecified adverse effect of drug or medicament, subsequent encounter Z88.1 Allergy status to other antibiotic agents

**ALL CAPS and bold** codes relate to *Clostridium* difficile; ***Bold italicized*** codes relate to candidiasis; Underlined codes related to adverse drug events.

**Table 2.** Attempts at capturing antibiotic ADEs via electronic data pull

	Attempt 1	Attempt 2	Attempt 3	Attempt 4	Attempt 5
Major changes from previous attempt	N/A	Narrowed outpatient clinic list to exclude those unlikely to prescribe antibiotics and/or manage ADEs AND excluded patients prescribed >2 non-antibiotic meds within past 14 days	Narrowed ICD-10 code list to only those listing antibiotic or medication ADE	Removed required criterion of antibiotic prescription within past 7 days	Added ICD-10 codes pertaining to C. diff and Candidiasis and reincluded criterion of antibiotic prescription within past 7 days
Included ICD-10 codes (see Table 2)	All	All	Underlined codes	Underlined codes	Underlined, bold italicized, and bold capitalized codes
Search criteria	Adult patients with VUMC outpatient clinic or ED encounter; prescribed antibiotics within 7 days prior to this visit	Same as previous	Same as previous	Adult patients with VUMC outpatient clinic or ED encounter	Adult patients with VUMC outpatient clinic or ED encounter; prescribed antibiotics within 7 days prior to this visit
Locations tracked	All VUMC outpatient clinics and ED	Select* VUMC outpatient clinics and ED	Same as previous	Same as previous	Same as previous
Duration of data pull	2 months	3 months	12 months	Same as previous	Same as previous
Total encounters	414	572	27	935	80
Results	<5% related to ADEs	<5% related to ADEs	>80% related to ADEs	∼10% related to ADEs	48.8% related to ADEs
Consensus	Results not specific enough	Results not specific enough	Criteria too narrow	Results not specific enough and data errors/mismatch in encounter dates	Criteria too narrow and results not specific enough

*Primary care clinics, walk-in clinics, medical sub-specialty, and surgical sub-specialty clinics.

### Manual EMR review to ascertain ADE ICD-10 codes and evaluate antibiotic appropriateness

After limited success, focus shifted to estimating antibiotic ADE incidence and identifying ICD-10 codes used by providers to document antibiotic ADEs via retrospective chart review of encounters within 14 days of antibiotic prescription from eight VUMC WICs (8/1/23–10/31/23). Subsequent visit notes were manually reviewed for antibiotic ADEs, and billing codes were analyzed. For ADE cases, appropriateness of initial antibiotic choice, dose, and duration were assessed independently (NP, MZ) using national guidelines (Supplemental Table 1) and local antibiogram. Inappropriateness was defined as: (1) a non-first-line agent used without contraindication; or (2) a duration >3 days longer than recommended. Discordances were resolved by consensus.

### Prospective antibiotic ADE capture

Further, WIC clinicians responsible for patient phone calls or return visits at these clinics were asked to voluntarily complete a REDCap survey (Supplemental Figure 1) when patients called or returned with antibiotic ADE symptoms within 14 days of receiving antibiotics at any outpatient VUMC clinic or ED (11/1/23–4/30/24). All REDCap entries were chart reviewed for antibiotic appropriateness using the same criteria described above.

## Results

### Electronic data extraction using ICD-10 codes

Results from the attempted electronic capture of patient encounters related to suspected antibiotic ADEs varied (Table 2). A range of 27–935 potential antibiotic ADE encounters were identified, of which <5% up to 81.5% were true antibiotic ADE encounters.

### Retrospective chart review

From retrospective chart review of 1059 unique patient encounters (8/1/23–10/31/23), 69 (6.5%) had antibiotic ADEs documented in subsequent visits (Supplemental Figure 2). Antibiotic ADEs were the primary reason for the visit in 39 visits (56.5%), and secondary complaints in 30 visits (43.5%). Of the 69 encounters with documented antibiotic ADEs, 17 (24.6%) utilized a diagnosis/billing code that indicated a medication adverse event (Supplemental Table 2), more often in encounters when ADEs were the visit’s primary complaint.

The REDCap survey (11/1/23–4/30/24) recorded 45 patient calls and 3 return visits with antibiotic ADEs, 0.3% of 16,274 WIC visits with antibiotics during that same time.

### Antibiotic appropriateness

Of the 69 retrospective chart reviews and 48 REDCap survey antibiotic ADEs, 17/69 (24.6%) and 6/48 (12.5%) antibiotic prescriptions were not indicated. Of the 94/117 (80.3%) combined that were indicated, 31/94 (33.0%) prescriptions had a wrong antibiotic selection (14/31, 45.2%), dose (11/31, 35.5%), or duration (7/31, 22.6%) (Supplemental Figure 3).

The most frequently reported side effect was gastrointestinal upset (43/11, 36.8%), followed by vaginal candidiasis (28/117, 23.9%) and rash (27/117, 23.1%). Less common side effects included facial/throat swelling, oral candidiasis, headache, fatigue, dizziness, and mouth sores/ulcers.

For our REDCap survey cohort, 13/48 (27.1%) reported no improvement in symptoms with antibiotic use.

## Discussion

Outpatient antibiotic ADEs are not uncommon. Sustainable, efficient tracking remains challenging. Antibiotic-related billing codes (Table 1, underlined codes) are underused, and repeated attempts to identify ADEs electronically using ICD-10 codes—including those for symptoms or related infections like candidiasis—were unsuccessful.

Retrospective review of patients who received antibiotics revealed challenges in building a reliable electronic auditing tool. Among 69 encounters with documented antibiotic ADEs, 17 (24.6%) used ADE-related billing codes. Most used carryover diagnoses or symptomatic codes (eg, rash) without association with suspected causative medications, highlighting difficulties in using symptomatic ICD-10 codes for antibiotic ADE capture. Voluntary REDCap survey reporting accurately captured patient calls but grossly underestimated incidence and added significant documentation burden, limiting its sustainability and overall utility.

Few studies have successfully used ICD-10-based algorithms to track outpatient antibiotic ADEs in adults and highlight struggles with limited specificity,^7^ and/or low sensitivity, especially for certain infections.^8^ Hence, while ICD-10 codes can support monitoring of antibiotic use, when used alone, they lack the accuracy to reliably capture outpatient antibiotic ADEs in adults.

Outpatient ADEs are likely under-reported and/or under-documented. The discrepancy between ADEs documented as secondary complaints and those reported by phone suggests patients are more likely to call to report ADEs rather than schedule visits. Implementing a standardized system for logging ADE-related patient calls could improve capture, but most EMRs currently lack associated diagnosis codes, limiting electronic tracking. Emerging AI tools like natural language processing (NLP) offer promise in identifying ADEs from unstructured notes;^9,10^ however, standardization and integration challenges persist.

Notably, 23/48 (47.9%) of surveyed patients reported partial or no symptom improvement with antibiotic prescription, supporting the need for ongoing outpatient AS.

### Limitations

Limitations included limited generalizability given single-center design including only adult encounters in WICs and short-term antibiotic ADEs occurring within 7–14 days of initiation. The REDCap survey participation was voluntary, likely leading to missed in-person encounters from busy WIC providers; however, three clinicians (EN, RT, AZ) handled all WIC phone calls, ensuring high capture of ADEs via calls. Survey responses involved some provider clinical judgment, which could misattribute complaints to antibiotics rather than other causes. Overall, these limitations more likely led to under-estimation of antibiotic ADE’s. Further, due to its exploratory nature, ICD-10 code search criteria and time frames varied across extraction attempts limiting direct comparison of quantitative yield across different strategies and their yield compared to the rate estimated by chart review. Finally, our strict criteria for antibiotic appropriateness, especially regarding duration, may have misclassified some longer courses as inappropriate.

## Conclusion

Despite limitations, including likely under capture of antibiotic ADEs, our study showed that antibiotic ADEs are not uncommon, affecting at least >1 in every 20 outpatients who receive antibiotics, but lack of accurate ICD-10-code-based methods to capture antibiotic ADEs limits surveillance and intervention. Utilizing voluntary reporting increased capture of antibiotic ADE patient calls but was not comprehensive nor efficient. There is a critical need for innovative approaches and advanced tools to accurately identify and extract antibiotic ADE data to improve surveillance and reporting to support better patient safety and clinical outcomes.

## Supporting information

10.1017/ash.2026.10356.sm001 Patel et al. supplementary material Patel et al. supplementary material

## Supplementary material

The supplementary material for this article can be found at https://doi.org/10.1017/ash.2026.10356.

## Research transparency and reproducibility

The datasets generated and analyzed during this study are not publicly available due to patient privacy and institutional policy restrictions. Deidentified data may be available from the corresponding author upon reasonable request and with appropriate institutional approvals. No custom code was used in this study.
