# Supplementary material for: Uncharted territory: assessing antibiotic adverse drug events from walk-in clinics at an academic healthcare system
Source: Antimicrob Steward Healthc Epidemiol. 2026 Apr 17;6(1):e107. doi: 10.1017/ash.2026.10356 (PMC13104572; doi:10.1017/ash.2026.10356)
Supplement: Patel et al. supplementary material [file S2732494X26103568sup001.zip › Supplementary material/Supplemental Table 1 and 2. Final.docx]

**Supplemental Table 1 - Guidelines Utilized to Determine Antibiotic Appropriateness**

| **Diagnosis** | **Organization/Association** | **Title of Guideline** | **URL** |
| --- | --- | --- | --- |
| Acute rhinosinusitis | Centers for Disease Control and Prevention | Outpatient Clinical Care for Adults | <https://www.cdc.gov/antibiotic-use/hcp/clinical-care/adult-outpatient.html?CDC_AAref_Val=https://www.cdc.gov/antibiotic-use/clinicians/adult-treatment-rec.html> |
|  | Infectious Diseases Society of America | IDSA Clinical Practice Guideline for Acute Bacterial Rhinosinusitis in Children and Adults | <https://academic.oup.com/cid/article/54/8/e72/367144> |
| Acute uncomplicated bronchitis | Centers for Disease Control and Prevention | Outpatient Clinical Care for Adults | <https://www.cdc.gov/antibiotic-use/hcp/clinical-care/adult-outpatient.html?CDC_AAref_Val=https://www.cdc.gov/antibiotic-use/clinicians/adult-treatment-rec.html> |
| Nonspecific upper respiratory tract infection | Centers for Disease Control and Prevention | Outpatient Clinical Care for Adults | <https://www.cdc.gov/antibiotic-use/hcp/clinical-care/adult-outpatient.html?CDC_AAref_Val=https://www.cdc.gov/antibiotic-use/clinicians/adult-treatment-rec.html> |
| Pharyngitis | Centers for Disease Control and Prevention | Outpatient Clinical Care for Adults | <https://www.cdc.gov/antibiotic-use/hcp/clinical-care/adult-outpatient.html?CDC_AAref_Val=https://www.cdc.gov/antibiotic-use/clinicians/adult-treatment-rec.html> |
| Acute uncomplicated cystitis | Centers for Disease Control and Prevention | Outpatient Clinical Care for Adults | <https://www.cdc.gov/antibiotic-use/hcp/clinical-care/adult-outpatient.html?CDC_AAref_Val=https://www.cdc.gov/antibiotic-use/clinicians/adult-treatment-rec.html> |
| Acute uncomplicated cystitis and pyelonephritis | Infectious Diseases Society of America and the European Society for Microbiology and Infectious Diseases | International Clinical Practice Guidelines for the Treatment of Acute Uncomplicated Cystitis and Pyelonephritis in Women: A 2010 Update by the Infectious Diseases Society of America and the European Society for Microbiology and Infectious Diseases | <https://academic.oup.com/cid/article/52/5/e103/388285?login=true> |
| Asymptomatic bacteriuria | Infectious Diseases Society of America | Clinical Practice Guideline for the Management of Asymptomatic Bacteriuria: 2019 Update by IDSA | <https://www.idsociety.org/practice-guideline/asymptomatic-bacteriuria/> |
| Community acquired pneumonia | American Thoracic Society/Infectious Diseases Society of America | ATS/IDSA Guidelines for Diagnosis and Treatment of Adults with Community-acquired Pneumonia | <https://www.idsociety.org/practice-guideline/community-acquired-pneumonia-cap-in-adults> |
| Influenza | Infectious Diseases Society of America | Clinical Practice Guidelines for the Diagnosis, Treatment, Chemoprophylaxis, and Institutional Outbreak Management of Seasonal Influenza: 2018 Update by IDSA | <https://www.idsociety.org/practice-guideline/influenza/#RecommendationsAbridged> |
| Cellulitis | Centers for Disease Control and Prevention | Clinical Guidance for Group A Streptococcal Cellulitis | <https://www.cdc.gov/group-a-strep/hcp/clinical-guidance/cellulitis.html> |
|  | Infectious Diseases Society of America | Clinical Practice Guidelines for the Diagnosis and Management of Skin and Soft Tissue Infections: 2014 Update by IDSA | <https://www.idsociety.org/practice-guideline/skin-and-soft-tissue-infections> |
| Infectious diarrhea | Infectious Diseases Society of America | IDSA 2017 Clinical Practice Guidelines for the Diagnosis and Management of Infectious Diarrhea | <https://www.idsociety.org/practice-guideline/infectious-diarrhea/> |
| Diverticulitis | American College of Physicians | Diagnosis and Management of Acute Left-Sided Colonic Diverticulitis: A Clinical Guideline From the American College of Physicians | <https://www.acpjournals.org/doi/full/10.7326/M21-2710?rfr_dat=cr_pub++0pubmed&url_ver=Z39.88-2003&rfr_id=ori%3Arid%3Acrossref.org> |
|  | American Gastroenterological Association | American Gastroenterological Association Institute Guideline on the Management of Acute Diverticulitis | <https://www.sciencedirect.com/science/article/pii/S0016508515014328?via%3Dihub#sec1> |
|  | The American Society of Colon and Rectal Surgeons | The American Society of Colon and Rectal Surgeons Clinical Practice Guidelines for the Treatment of Left-Sided Colonic Diverticulitis | <https://journals.lww.com/dcrjournal/Fulltext/2020/06000/The_American_Society_of_Colon_and_Rectal_Surgeons.6.aspx> |

**Supplemental Table 2: Billing codes utilized (drug/allergy-related)**

| **ICD-10 Code** | **Code Description** |
| --- | --- |
| T88.7 | Adverse effect of drug (2) |
| T78.4 | Allergic disorder |
| L27.0 | Allergic drug rash |
| Z88.3 | Allergic drug rash due to anti-infective |
| T78.40 | Allergic reaction (2) |
| Z88.8 | Allergic reaction to drug |
| T36.0 | Amoxicillin-induced allergic drug rash |
| T78.3 | Angioedema with urticaria due to drug |
| K52.1 | Antibiotic-associated diarrhea |
| Z88.8 | Medication intolerance (2) |
| T88.7 | Medication reaction |
| T88.7 | Medication side effect |
| K20.8 | Pill esophagitis |
| L27.0 | Rash due to allergy |

Number in parenthesis indicates number of times code was utilized
