# Supplementary figures and images for: Uncharted territory: assessing antibiotic adverse drug events from walk-in clinics at an academic healthcare system
Source: Antimicrob Steward Healthc Epidemiol. 2026 Apr 17;6(1):e107. doi: 10.1017/ash.2026.10356 (PMC13104572; doi:10.1017/ash.2026.10356)

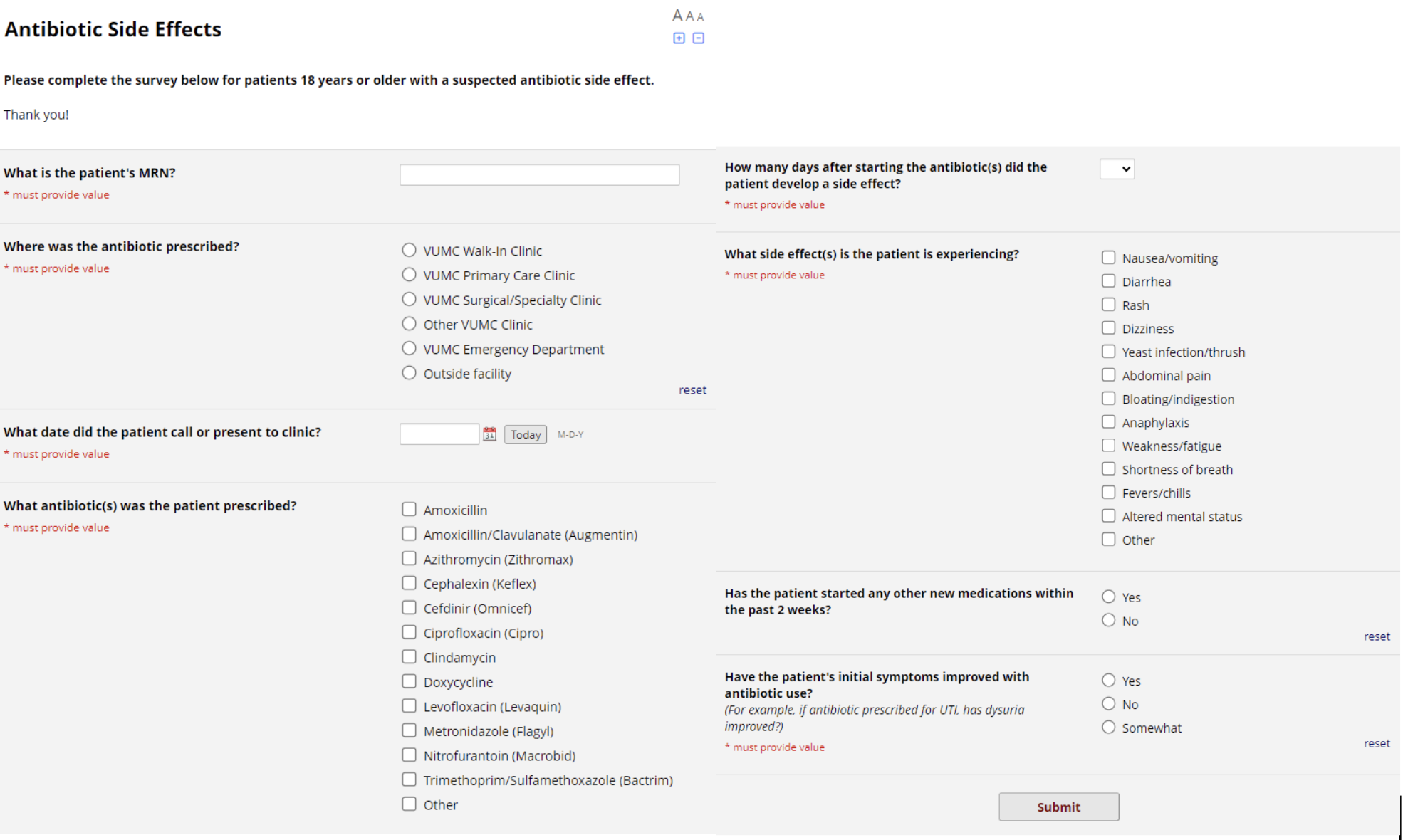

Supplement: Patel et al. supplementary material [file S2732494X26103568sup001.zip › Supplementary material/Supplemental Figure 1 REDCap.tiff]

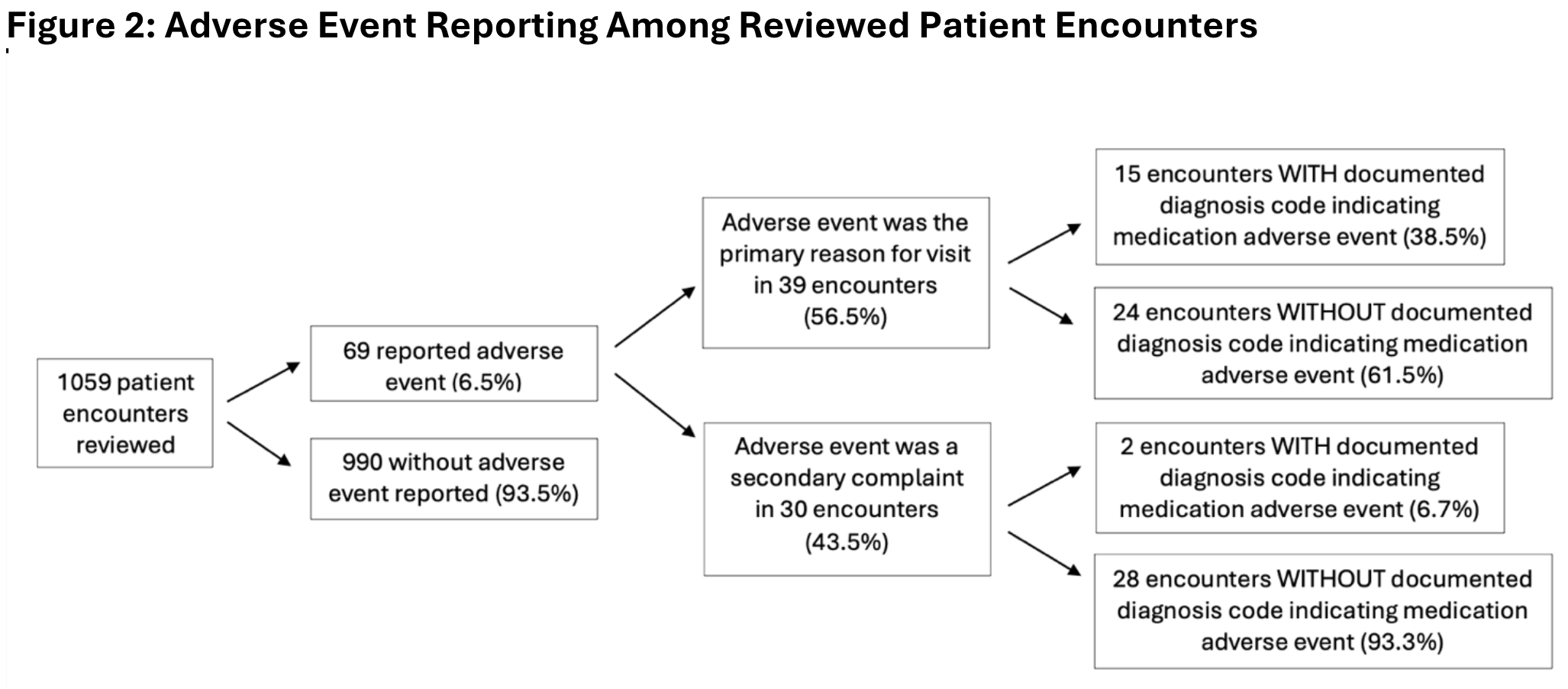

Supplement: Patel et al. supplementary material [file S2732494X26103568sup001.zip › Supplementary material/Supplemental Figure 2 ADEs.tiff]

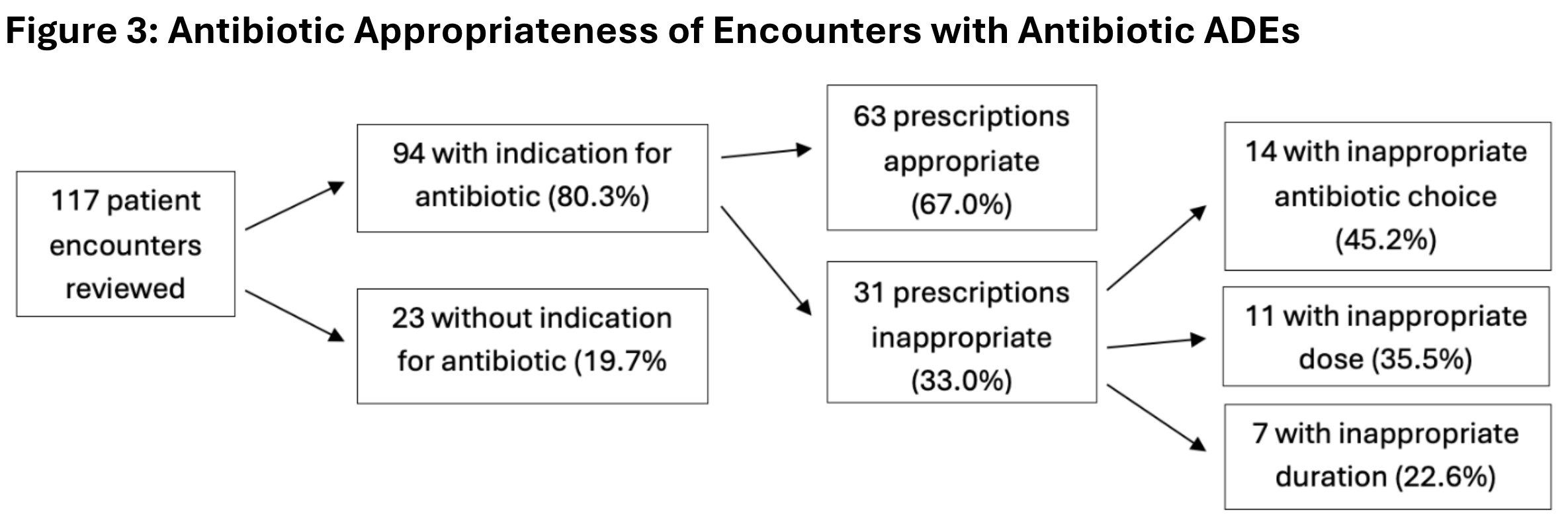

Supplement: Patel et al. supplementary material [file S2732494X26103568sup001.zip › Supplementary material/Supplemental Figure 3 Abx Appropriateness.tiff]
